# Dengue virus infection induces autophagy: an *in vivo* study

**DOI:** 10.1186/1423-0127-20-65

**Published:** 2013-09-08

**Authors:** Ying-Ray Lee, Hsuan-Yun Hu, Szu-Han Kuo, Huan-Yao Lei, Yee-Shin Lin, Trai-Ming Yeh, Ching-Chuan Liu, Hsiao-Sheng Liu

**Affiliations:** 1 Department of Medical Research, Chiayi Christian Hospital, Chiayi, Taiwan; 2 Department of Microbiology and Immunology, College of Medicine, National Cheng Kung University, Tainan, Taiwan; 3 Department of Medical Laboratory Science and Biotechnology, College of Medicine, National Cheng Kung University, Tainan, Taiwan; 4 Department of Pediatrics, College of Medicine, National Cheng Kung University, Tainan, Taiwan; 5 Center of Infectious Disease and Signaling Research, College of Medicine, National Cheng Kung University, Tainan, Taiwan

**Keywords:** Dengue virus, Autophagy, Suckling mice

## Abstract

**Background:**

We and others have reported that autophagy is induced by dengue viruses (DVs) in various cell lines, and that it plays a supportive role in DV replication. This study intended to clarify whether DV infection could induce autophagy *in vivo*. Furthermore, the effect of DV induced autophagy on viral replication and DV-related pathogenesis was investigated.

**Results and conclusions:**

The physiopathological parameters were evaluated after DV2 was intracranially injected into 6-day-old ICR suckling mice. Autophagy-related markers were monitored by immunohistochemical/immunofluorescent staining and Western blotting. Double-membrane autophagic vesicles were investigated by transmission-electron-microscopy. DV non-structural-protein-1 (NS1) expression (indicating DV infection) was detected in the cerebrum, medulla and midbrain of the infected mice. In these infected tissues, increased LC3 puncta formation, LC3-II expression, double-membrane autophagosome-like vesicles (autophagosome), amphisome, and decreased p62 accumulation were observed, indicating that DV2 induces the autophagic progression *in vivo*. Amphisome formation was demonstrated by colocalization of DV2-NS1 protein or LC3 puncta and mannose-6-phosphate receptor (MPR, endosome marker) in DV2-infected brain tissues. We further manipulated DV-induced autophagy by the inducer rapamycin and the inhibitor 3-methyladenine (3MA), which accordingly promoted or suppressed the disease symptoms and virus load in the brain of the infected mice.

We demonstrated that DV2 infection of the suckling mice induces autophagy, which plays a promoting role in DV replication and pathogenesis.

## Background

DV is a positive single-strand RNA virus which belongs to the Flaviviridae family, and is composed of four serotypes (DV1 to 4). It is transmitted to vertebrate hosts via the mosquito vector *Aedes aegypti* and *A. albopictus*. DV causes over 390 million infections every year, and is one of the most important arboviruses causing human diseases [1]. The symptoms of DV infection range from mild dengue fever (DF) to life-threatening dengue haemorrhagic fever (DHF) and dengue shock syndrome (DSS). Generally speaking, DF is self-limited, but in a small proportion of people the disease may proceed to severe DHF/DSS. Diverse hypotheses have been proposed to explain the pathologic mechanism of DHF/DSS, but the findings remain contradictory.

Autophagy participates in the degradation of long-lived and aggregated protein as well as damaged organelles in the cytoplasm to maintain homeostasis. Autophagy is characterized by a double-membrane vesicle known as an autophagosome, which recruits cytoplasmic materials and fuses with lysosome for protein degradation [2]. Autophagy is classified as macroautophagy, microautophagy, and chaperon-mediated autophagy, depending on how it delivers the cargo to lysosome and its physiological function [3]. Aberrant autophagic activities lead to the pathogenesis of various diseases, including diabetes, neurodegeneration, heart disease, and cancers. Tian *et al.,* reported that autophagy was detected in brain sections of a GFP-LC3 transgenic mouse model after transient cerebral ischemia and demonstrated a relationship between autophagy and apoptosis [4,5]. Dengue virus infection induces apoptosis in various cell lines and clinical patient specimens [6]. However, dengue virus-induced apoptosis and its relationship with autophagy remain to be determined.

Beclin1 serves as a platform to recruit other regulatory molecules of C3-PI3K complex, including Atg14-like protein (Atg14L), UV irradiation resistance-associated gene (UVRAG), Bax-interacting factor-1 (Bif-1) and activating molecule in Beclin-1-regulated autophagy protein-1 (Ambra-1) [7-10]. During vesicle elongation, two ubiquitin-like conjugation systems are activated. First, Atg12 is covalently conjugated with Atg5 by E1-like enzyme Atg7 and E2-like enzyme Atg10. Second, Atg5 binds to Atg16L1, a coiled-coil domain-containing protein, to form a heterotrimeric complex, Atg5-Atg12-Atg16L1. This complex is responsible for the expansion of the phagophore and is dissociated from the membrane when autophagosome formation is completed. Microtubule-associated protein 1A/1B light chain 3 (LC3) (mammalian homologue of yeast Atg8) is initially cleaved by Atg4, a cysteine protease, followed by phosphatidylethanolamine (PE) modification on the carboxyl terminus of the cleaved LC3 [11]. The lipidated LC3 located on the membrane facilitates autophagosome maturation. The autophagosome may fuse with the endosome to form the amphisome or with the lysosome to form the autophagolysosome [12-14].

Autophagy is also involved in the host immunity against pathogen infection [15]. Autophagy acts as an anti-viral component of the innate immune system and is induced by the ligands of the toll-like receptors [16]. Furthermore, autophagy enhances the presentation of viral antigens by dendritic cells during the infection of Sendai and vesicular stomatitis viruses [17]. Autophagy can also function in the adaptive immune response by enhancing the presentation of antigen onto MHC class II molecules [18-20]. Autophagy not only plays an antiviral role, but also shows pro-viral functions [21,22]. Poliovirus, coxsackievirus B3, hepatitis C virus (HCV), coronavirus, enterovirus 71 and DV activate autophagy to elevate viral replication [23-28]. HCV uses autophagy for the early protein translation and suppresses the innate antiviral immunity [23,29]. The double membrane of the autophagosome may support poliovirus replication [30], and the autophagic machinery is utilized for the replication of coronaviruses [25,31]. DV infection increases autophagic activity to enhance viral replication, indicating the use of autophagosome as the docking site for viral replication complex or as the organelle for lipid metabolism to provide ATP energy for DV replication [25,32-35]. Autophagy induction by NS4A protein of DV prevents the infected cell from death and enhances viral replication [35]. While it is known that autophagy plays an important role in DV replication *in vitro*, the role of autophagy *in vivo* has not been reported. This study focused on autophagic activity, virus titer and pathogenesis in DV2 infection of the suckling mice.

## Methods

### Dengue virus and mice

The DV2 (strain PL046) was routinely maintained in *A. albopictus*-derived cell line C6/36 (purchased from ATCC). Breeder mice of the ICR strain were purchased from the National Laboratory Animal Center, Taiwan. The mice were maintained at the Animal Facility of National Cheng Kung University, Taiwan, and were manipulated according to the animal experiment guidelines of the National Science Council, Taiwan. Six-day-old suckling mice were inoculated intracerebrally with 2.5×10^5^ pfu of active or heat-inactivated DV2 or control Dulbecco’s Modified Eagle Medium (DMEM) (GIBCO BRL, Gaithersburg, MD, USA) containing 2% fetal bovine serum (FBS). The mice were sacrificed and perfused with isotonic saline containing EDTA. For plaque assay, the brain tissues were collected, weighed and homogenized in 1 ml of DMEM containing 2% FBS. The supernatant was collected by centrifugation at 8000 rpm for 15 min at 4°C and frozen at −70°C. For Western blot analysis, the brain tissues were homogenized with 1 ml of Radio-immunoprecipitation assay (RIPA) lysis buffer (50mM Tris, 150mM NaCl, 0.1% SDS, 0.5% sodium deoxycholate, 1% Triton X-100, PMSF, pH7-8) (Sigma-Aldrich, St. Louis, MO, USA). The supernatant was collected by centrifugation at 14000 rpm for 20 min at 4°C and frozen at −70°C. For IFA and IHC assays, the brain tissues were embedded in Tissue-Tek O. C. T. compound (Sankura Finetek, Torrance, CA, USA), frozen in liquid nitrogen and stored at −70°C. Serial coronal sections (5 μm) were cut on a cryostat (Leica CM1800, Heidelberg, Germany) and mounted on a silanized slide (Dako, Carpinteria, CA, USA).

### Plaque assay

BHK-21 cells were plated in a 12-well plate (9×10^4^ cells/well) and cultured in DMEM (GIBCO). After adsorption for 2 h with serially diluted virus solutions, the solution was replaced with fresh DMEM containing 2% FBS and 0.8% methyl cellulose (Sigma-Aldrich). At four days post-infection, the medium was removed and the cells were fixed and stained with the crystal violet solution consisting of 1% crystal violet, 0.64% NaCl, and 2% formalin for 1 h at room temperature (RT). Finally, the crystal violet was removed and the plate was washed with the tap water. The viral titer was determined by the plaque assay [25].

### Immunohistochemostry staining (IHC) and immunofluorescence assay (IFA)

Mice brain sections were fixed with acetone and blocked with 3% H_2_O_2_ (Merck, Darmstadt, Germany) in methanol. The sections were blocked with SuperBlock (SuperBlock blocking buffer in PBS; Thermo Scientific, Rockford, IL, USA) or Vector M.O.M mouse immunoglobulin G blocking reagent (Vector Laboratories, Burlingame, CA, USA) for 1 h at RT. The sections were further incubated with the anti-DV2-NS1 antibody (ab41632, Abcam, Cambridge, MA, USA), anti-autophagy LC3 antibody (AP1802a, Abgent, San Diego, CA, USA) or anti-Beclin 1 antibody (ab62472, Abcam), which was diluted in blocking buffer overnight at 4°C. The sections were further incubated with biotinylated secondary antibody (Dako, Glostrup, Denmark) for 1 h at RT and stained with AEC Substrate Chromogen (Dako). DAPI (4′-6-Diamidino-2-phenylindole; Sigma-Aldrich, St. Louis, MO, USA) was used to stain the nuclei of the brain cells. Subsequently, the slides were immersed in hematoxylin (Merck, Darmstadt, Germany) for counterstaining and then rinsed in tap water for 10 min. Finally, the slides were mounted with Dako Faramount Aqueous Mounting Medium (Dako).

For the immunofluorescence assay, the section after the primary antibody treatment was incubated with the secondary antibody conjugated with red (A11004, Invitrogen, Eugene, Oregon, USA) or green (A11008, Invitrogen) fluorescence. Subsequently, the slide was stained with Hoechst 33258 (Sigma-Aldrich). Finally, the sections were mounted and examined under a laser confocal scanning microscope (Olympus FluoView FV1000, CenterValley, PA, USA).

### Transmission electron microscopy

DV2 (2.5 x 10^5^ pfu/mouse) was intracranially injected into the brain of six-day-old ICR suckling mice. The clinical score and body weight were measured every day. Five mice were treated with live DV2, three were treated with heat inactive DV2 (iDV2), and three mice were treated with the culture medium. At five days post-infection, mice were sacrificed after anesthesia with 7% chloral hydrate followed by perfusion with 4% paraformaldehyde in 0.1M phosphate buffer (PB). After fixation, the cervical spinal cord was removed and kept in PB solution overnight at 4°C. The cervical cord was cut into 100 μm sections and fixed with 2.5% glutaraldehyde in 0.1 M cacodylate buffer (4% sucrose, 1 mM MgCl_2_ and 1 mM CaCl_2_) and post-fixed in 1% osmium tetroxide (Electron Microscopy Sciences, Hatfield, PA, USA). The sections were then dehydrated in a series dilutions of ethanol and embedded with LR White (Agar Scientific, Stansted, UK). Ultrathin sections were obtained using an ultramicrotome (Reichert-Jung, Heidelberg, Germany) and stained with saturated aqueous uranyl acetate (Electron Microscopy Sciences) and lead citrate (Electron Microscopy Sciences) at RT, and then investigated under a Hitachi H-7650 transmission electron microscope (Hitachi, Tokyo, Japan).

### Western blot analysis

Cells were cultured and infected with DV2, and the total cell extracts harvested at various time points were subjected to sodium dodecyl sulfate-polyacrylamide gel electrophoresis (SDS PAGE). The separated proteins in the gel were electrically transferred to a PVDF membrane (Millipore, Bedford, MA, USA), followed by hybridization with their corresponding specific primary antibodies (anti-LC3: PM036, MBL, Woburn, MA, USA; anti-Beclin 1: sc-11427, Santa Cruz, CA, USA; anti-p62: Santa Cruz; anti-β-actin: A5441, Sigma-Aldrich) and the secondary antibodies (Goat anti-mouse IgG peroxidase conjugated Ab: AP124P and Goat anti-rabbit IgG HRP conjugated: AP132P, Chemicon, Billerica, MA, USA). After incubation with enhanced chemiluminescence (ECL) solution (Millipore) for 1 min, the membrane was exposed to an X-ray film (Eastman Kodak, NY, USA).

### Statistical analysis

Data are presented as the mean ± standard deviation. Differences between the test and control groups were analyzed by the Student’s *t* test using the Prism software. Significance was set at *p* < 0.05 (*), *p* < 0.01 (**) and *p* < 0.05 (***).

## Results

### Dengue virus type 2 infection of the ICR suckling mice causes physiopathological changes

**Figure 1:**
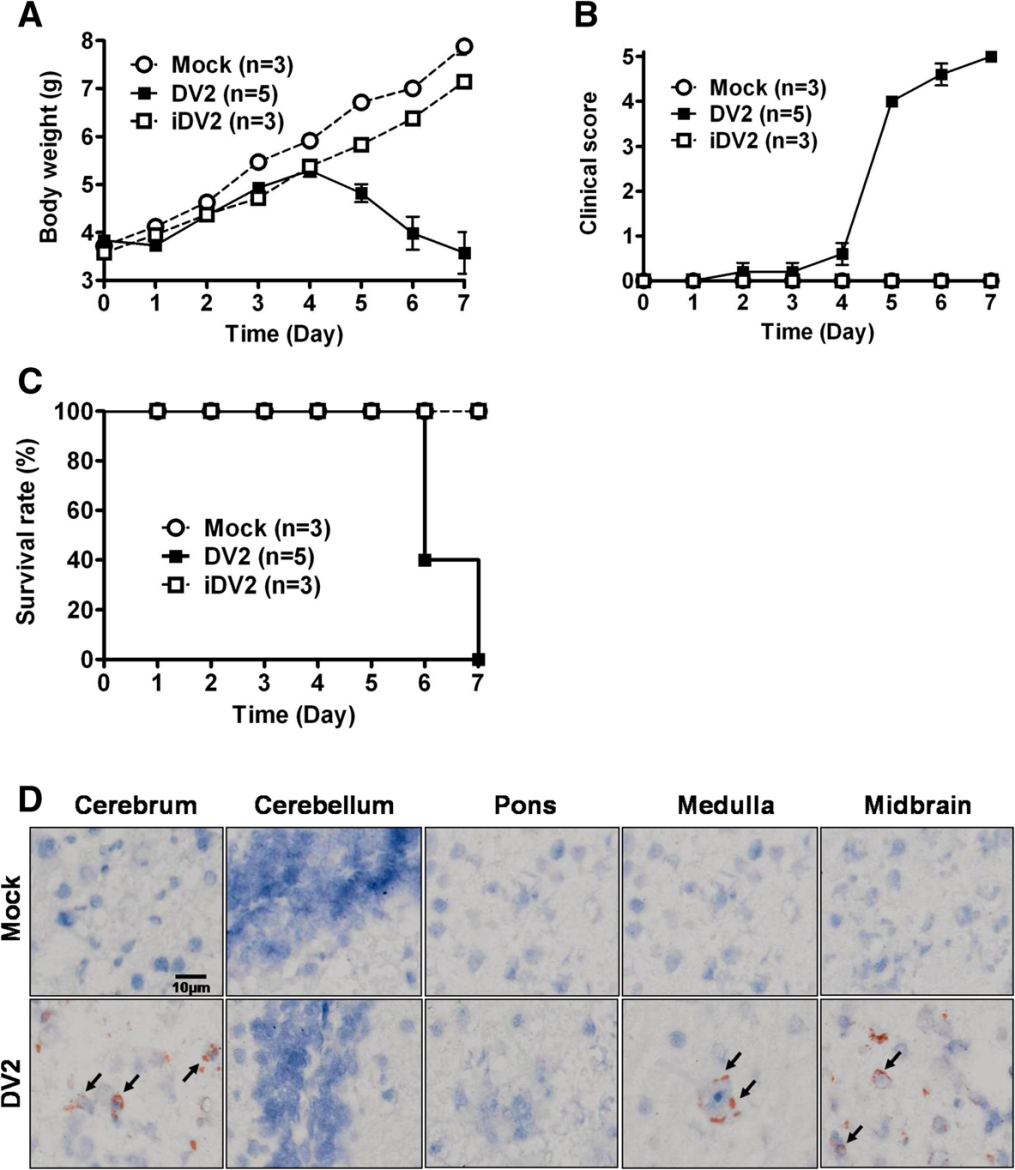
**Dengue-2 virus infection induces physiopathological changes of the ICR mice.** Six-day-old ICR suckling mice were intracranially inoculated with DV2 (2.5×10^5^ pfu/mouse). The **(A)** body weight **(B)** clinical score and **(C)** survival rate were monitored daily after inoculation. **(D)** The mice were sacrificed at day 5 p.i. The brain tissues were cryosectioned and DV2-NS1 expression in various regions of the mouse brain was detected with anti-DV NS1 antibody by IHC staining. Arrow points NS1 labeling. Disease symptoms were scored as follows: 0 for healthy; 1 for lightly sick (losing weight and ruffled hair); 2 for slow-moving and reduced mobility; 3 for moving with difficulty and anterior limb or posterior limb weakness; 4 for paralysis and mortally ill; 5 for death. Mock group was treated with 2% FBS/DMEM and iDV2 group was the virus subjected to heat treatment at 56°C for 30 min. The numbers of mice in each group were from 3 to 5. Data were analyzed using the Student’s *t* test. Significance was set at *p* < 0.05 (*), *p* < 0.01 (**), *p* < 0.05 (***).

We and others have demonstrated that dengue virus infection of various human cell lines induces autophagy, which further promotes virus replication [25,33,36]. In order to establish the role of DV2 infection in the induction of autophagy as well as its roles in DV replication and DV-related pathogenesis *in vivo*, DV2 or UV-inactivated DV2 (iDV2) (2.5×10^5^ pfu/mouse) was intracranially injected into six-day-old ICR suckling mice. The body weight, clinical score, and survival rate of the infected mice were measured daily for seven days. The body weight of DV2-infected mice was greatly decreased from day 5 to day 7 post infection (p.i) as compared to the mock-infected and iDV2-infected groups (Figure 1A). The disease symptoms progressed from mild sickness (losing weight and ruffled hair) at days 2 and 3 to severe paralysis and mortally ill at day 5 p.i., and mice started to die in the DV2-infected group, whereas no deaths were noted in the mock- and iDV2-treated groups at day 6 p.i. (Figure 1B). The survival rate in the DV2-infected mice was 40% at day 6 p.i and all of the mice died at day 7 p.i., but no mice died in the other two groups (Figure 1C). To verify that the abovementioned disease symptoms were indeed caused by dengue virus infection, NS1 protein, an indicator of DV infection, was detected by anti-NS1 antibody in the brain tissues, including the cerebrum, medulla and midbrain of the infected mice, but no NS1 antigen was seen in the cerebellum and pons (Figure 1D, arrow). Altogether, DV2 was shown to be capable of infecting the brain tissue of the ICR mice, and of causing severe disease symptoms leading to death.

### Dengue virus induces amphisome and autophagosome formation as well as autophagic flux in the brain of infected mice

**Figure 2:**
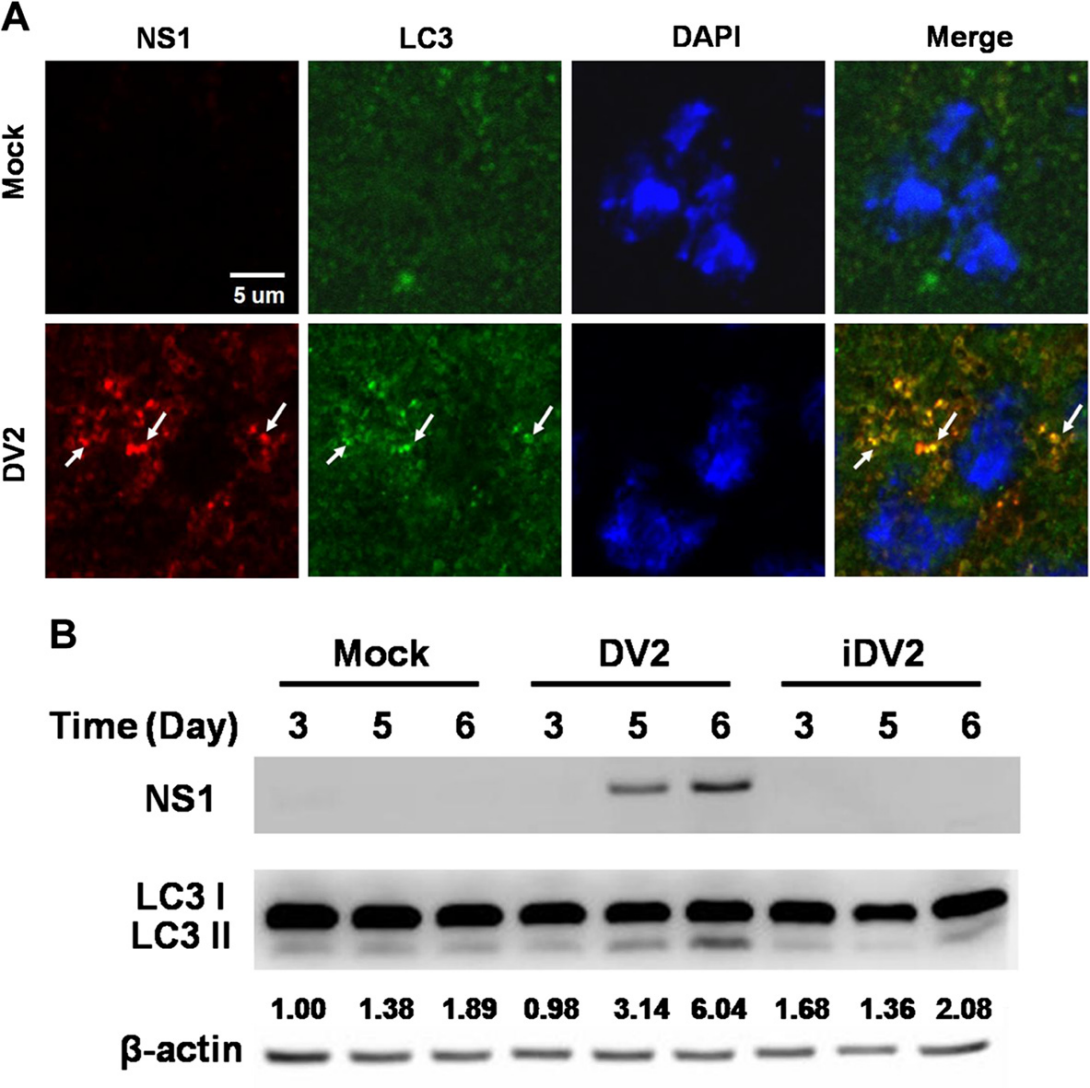
**Dengue-2 virus infection induces LC3 puncta formation and increases LC3 II expression in the mouse brain tissue.** As in Figure 1, DV2 was injected into the six-day-old suckling mice. **(A)** At day 5 p.i., the brain tissues were sectioned and the expression of DV2-NS1 and LC3 in the mouse brain tissue was determined with anti-DV NS1 and anti-LC3 antibodies by IFA staining under the confocal microscope (Olympus FluoView 1000). DAPI was used to stain the nucleus of the cell. Arrow points indicate the colocalization of NS1 and LC3. **(B)** The brain tissues of the mice used in **(A)** were harvested and total protein lysate was collected at various times p.i. and analyzed by Western blotting to measure the expression of DV2-NS1 and LC3 with the anti-DV NS1 and anti-LC3 antibodies. The numbers under each band are the quantification of LC3II band intensity after normalization with β-actin.

**Figure 3:**
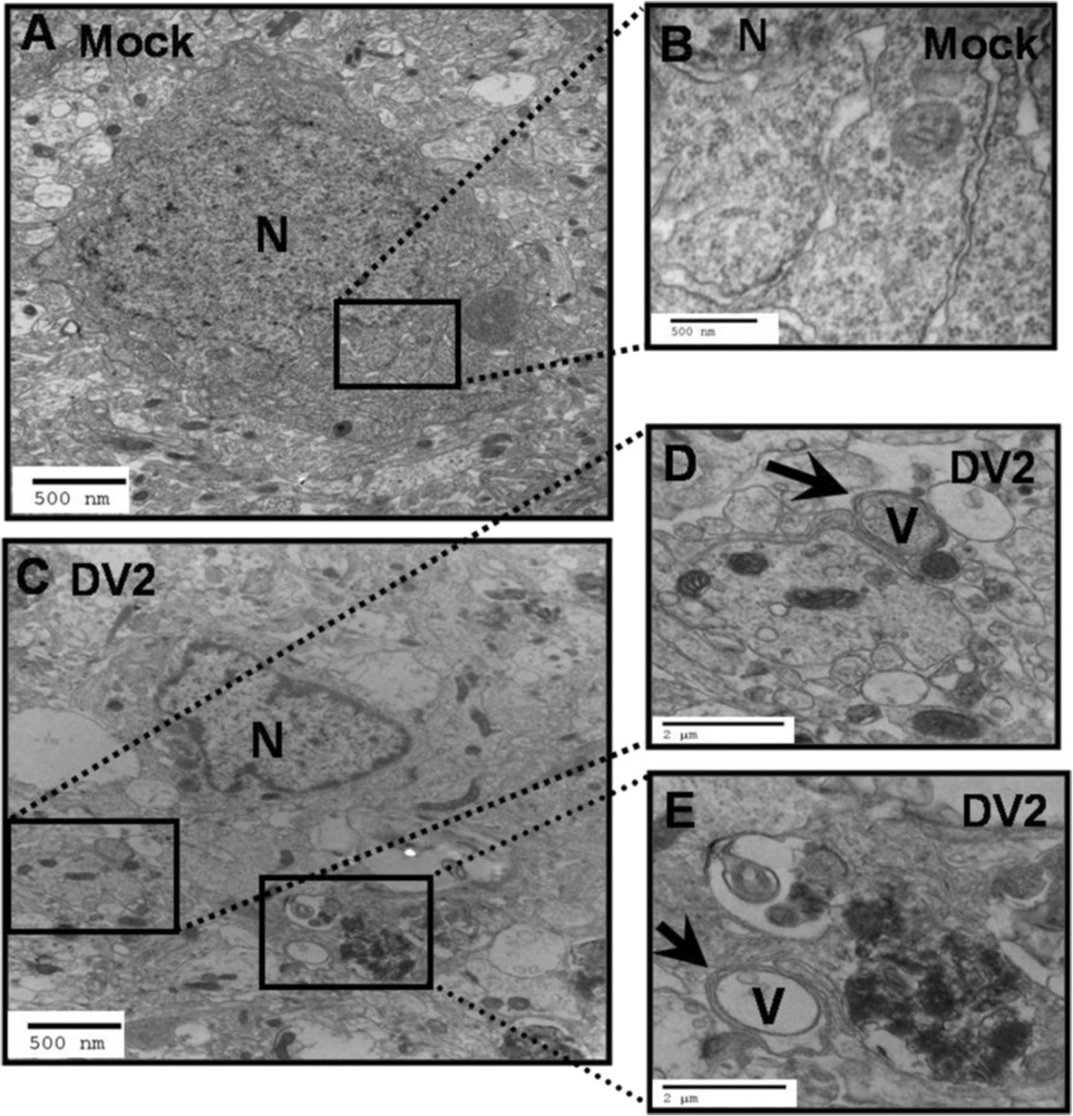
**DV2 infection of mice brain induces double-membrane vesicle formation.** The same treatment as in Figures 1 and 2 was conducted in this study. The brain tissues were collected and investigated by TEM. **(A)** Mock group was treated with DMEM containing 2% FBS. The enlargement of the square region in panel **(A, 12000X)** is shown in **(B, 25000X)**. **(C)** DV2-infected brain tissue of the suckling mice. The enlargements of the square regions in panels **(C, 12000X)** are shown in **(D, 25000X)** and **(E, 25000X)**, respectively. Arrow points indicate the double-membrane vesicles representing the autophagosome-like vesicles. N: nucleus; V: double-membrane vesicle.

**Figure 4:**
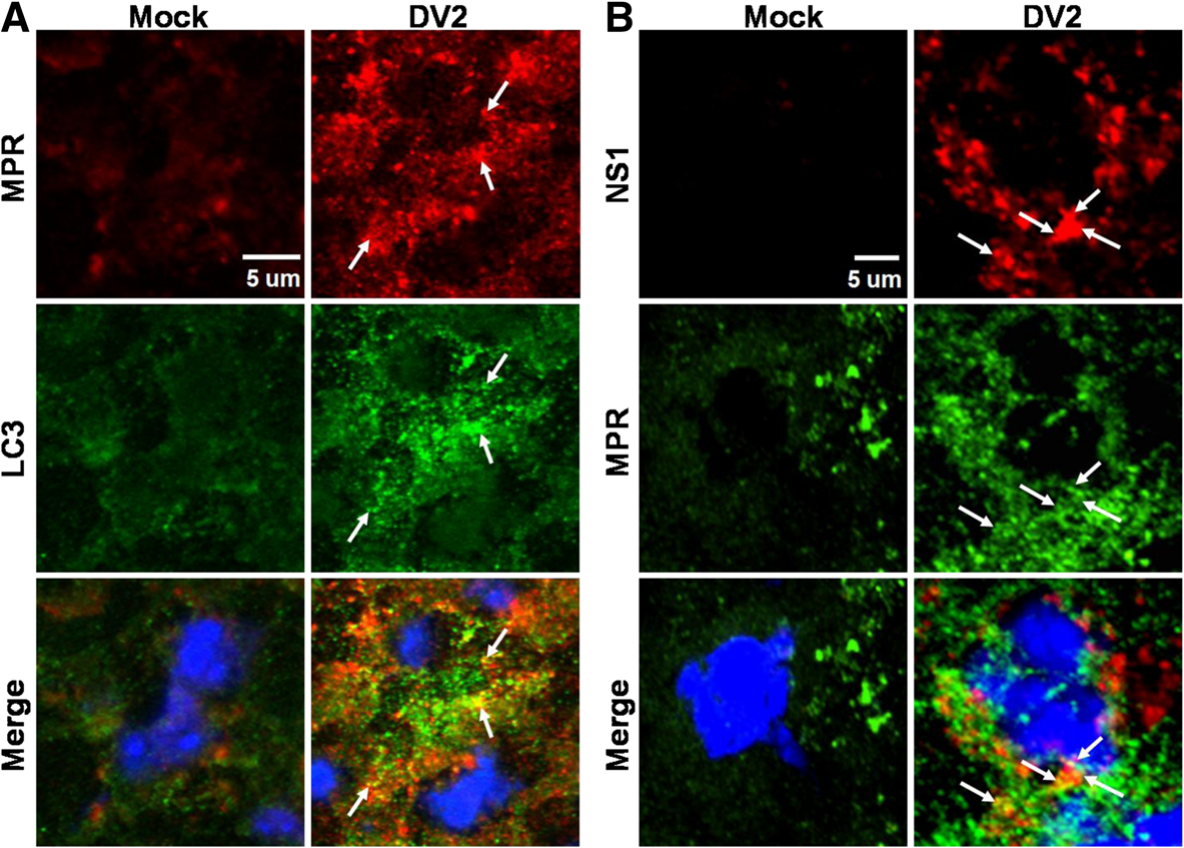
**DV2 infection of mice brain induces amphisome formation.** The same treatment used in the above figures was conducted. The expression of NS1, the endosome marker MPR, and the autophagosome marker LC3 protein in the sections of the mice brain tissue was shown by IFA staining under a confocal microscope using specific antibodies followed by secondary fluorescent conjugation. The DAPI was used to indicate the nucleus of the cells. **(A)** colocalization of LC3 (green) and MPR (red); **(B)** colocalization of NS1 (red) and MPR (green). Arrow points indicate colocalization of LC3 and MPR or NS1 and MPR.

The aforementioned data showed that DV-NS1 antigen was detected in the brain tissues of the infected mice (Figure 1). In order to establish that autophagy was induced in these DV2-infected mice, aggregation (puncta formation) of endogenous LC3 protein (a marker of autophagy) and DV2 NS1 expression in the brain tissue were assessed. The increased green fluorescent LC3 puncta, representing autophagosome formation, and the increased red fluorescent, representing DV-NS1 expression, were detected in the DV2-infected mice brain as compared to levels found in the mock-infected mice (Figure 2A, lower panels). Furthermore, colocalization of green LC3 puncta and red DV2-NS1 was seen in the brain sections of the infected mice (Figure 2A, arrow), suggesting that autophagosome formation was induced and that NS1, as a component of the replication complex working in concert with autophagosomes, may participate in DV replication. The expression levels of DV2 NS1 and LC3 II protein (a marker of autophagosome formation) in the brain were increased only in DV2-infected mice at days 5 and 6 p.i. compared with mock-and iDV2-infected mice. This result further supports the finding showing that DV induces autophagy *in vivo* (Figure 2B), and these phenomena were not seen in the heat-inactivated DV2 infection and mock control groups. This finding indicates that autophagy induction requires active DV infection and/or replication. Beclin 1, a coiled-coil protein, interacts with Bcl-2 and plays a critical role during autophagy progression [37]. However, in this *in vivo* study, Beclin 1 expression was not changed either in infected or in mock-infected mice brains by Western blotting at days 3, 5 and 6 p.i. and IHC assay at day 5 p.i. (Additional file 1: A and B, arrow). In summary, the role of Beclin 1 in DV2-induced autophagy requires further confirmation. The double-membrane autophagosome-like vesicles (V) were detected in the brain tissue of DV2-infected mice (Figure 3C, 3D and 3E, arrow), but not in the mock-infected group (Mock) (Figure 3A and 3B) under transmission electron microscopy. This double-membrane ultrastructure further supports the result showing that DV induces autophagy *in vivo*. We further detected the colocalization of green LC3 puncta and red MPR in the brain tissue of DV2-infected mice, indicating fusion of endosome with autophagosome to form amphisome (Figure 4A, arrow). We also observed the colocalization of DV2 NS1 (red) and MPR (green fluorescence, a marker of endosome) protein in brain sections under fluorescent microscopy, indicating that DV2 existed in the endosome (Figure 4B, arrow), which is consistent with an *in vitro* investigation by Panyasrivanit *et al.* that showed dengue virus was recruited into the endosome, which was then fused with the autophagosome to form amphisomes [36]. The data described above demonstrate that DV2 infection *in vivo* can induce autophagy and amphisome formation.

**Figure 5:**
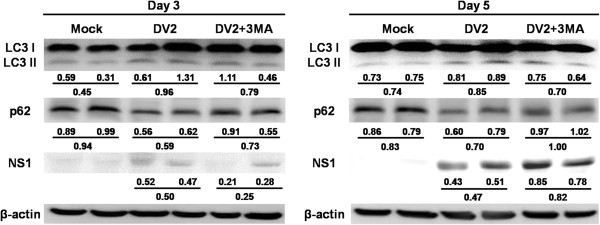
**Autophagic flux is induced in the brain tissue of DV2-infected mice.** The six-day-old ICR suckling mice were pre-treated with 3-MA (80μg/g) or PBS by intracranial inoculation followed by DV2 (2.5 x 10^5^ pfu/mouse) inoculation 2 h later. The brain tissues of two mice from each group were harvested and total protein lysate was collected at day 3 and day 5 p.i. and analyzed by Western blotting to measure the expression levels of LC3I, LC3II, p62, and DV2-NS1. The numbers under each band are the quantification and the mean of the intensity of LC3II, p62, and NS1 bands after normalization with β-actin.

The polyubiquitin-binding protein p62/SQSTM1 interacts with the ubiquitinated cargo protein followed by binding with LC3, and then is transported into the autophagosomes for degradation. The degradation of p62 has been used as an indicator of autophagic progression from autophagosome to autophagolysosome. To further confirm that DV2 infection indeed induces autophagic progression *in vivo*, the kinetics of LC3, p62, and NS1 expression level in two representative mice were evaluated at days 3 and day 5 p.i. by Western blotting. When the expression level of LC3II was induced at days 3 and 5 p.i., the expression of p62 was decreased in the DV2-infected group compared to that of the mock-infected group (Figure 5, 0.59 vs. 0.94 and 0.70 vs. 0.83, respectively). Furthermore, 3-MA was used to block DV2-induced autophagy, and the degradation level of p62 was reversed in the DV2 infection group at days 3 and day 5 p.i. (Figure 5, 0.73 vs. 0.59 at day 3, and 1.00 vs. 0.7 at day 5). These data indicate that an autophagic flux was induced during DV2 infection *in vivo*. Taken together, these findings demonstrate formation of amphisome and autophagosome, as well as the autophagic flux were induced in the DV2-infected brain tissues of the mice.

### Regulation of autophagy affects DV-related pathogenesis of suckling mice during dengue virus infection

**Figure 6:**
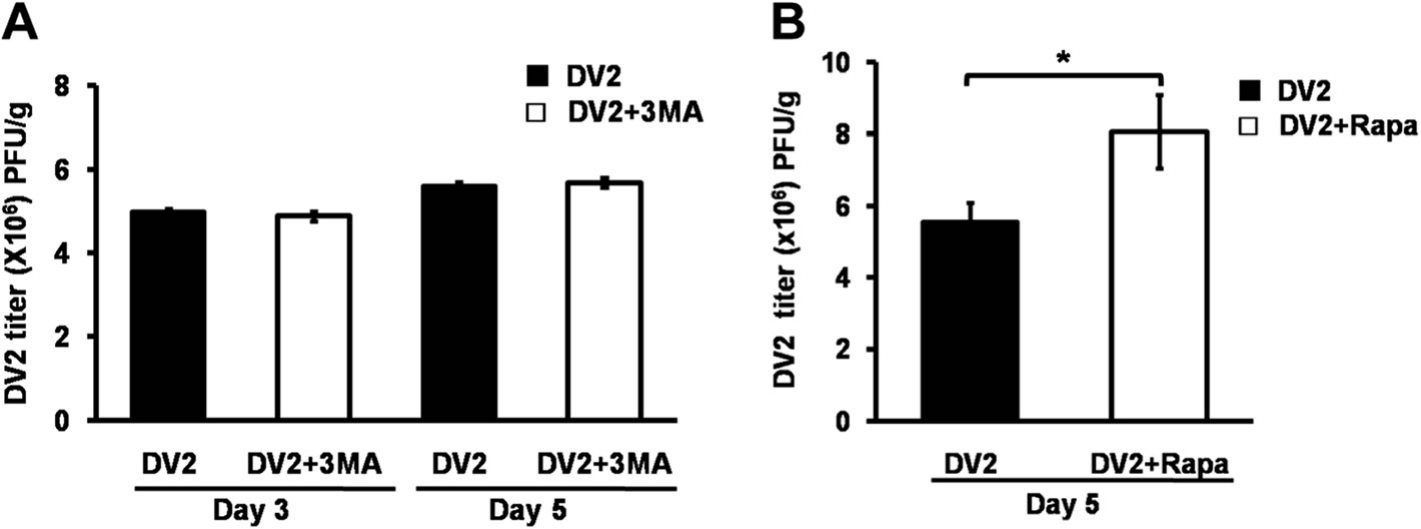
**The DV2 titer was affected by manipulating autophagy activity in DV2-infected mice brain.** Six-day-old ICR suckling mice were pre-treated with 3-MA (80μg/g), rapamycin (0.15μg/g), or PBS by intracranial inoculation, followed by DV2 (2.5 x 10^5^ pfu/mouse) inoculation 2 h later. Plaque assay was conducted to measure the viral titer in DV2-infected mice brain tissue at day 3 and day 5 p.i. in **(A)** together with 3-MA (DV2+3MA) and at day 5 p.i. in **(B)** together with rapamycin (DV2+Rapa). PBS-treated group is shown as DV2.

**Figure 7:**
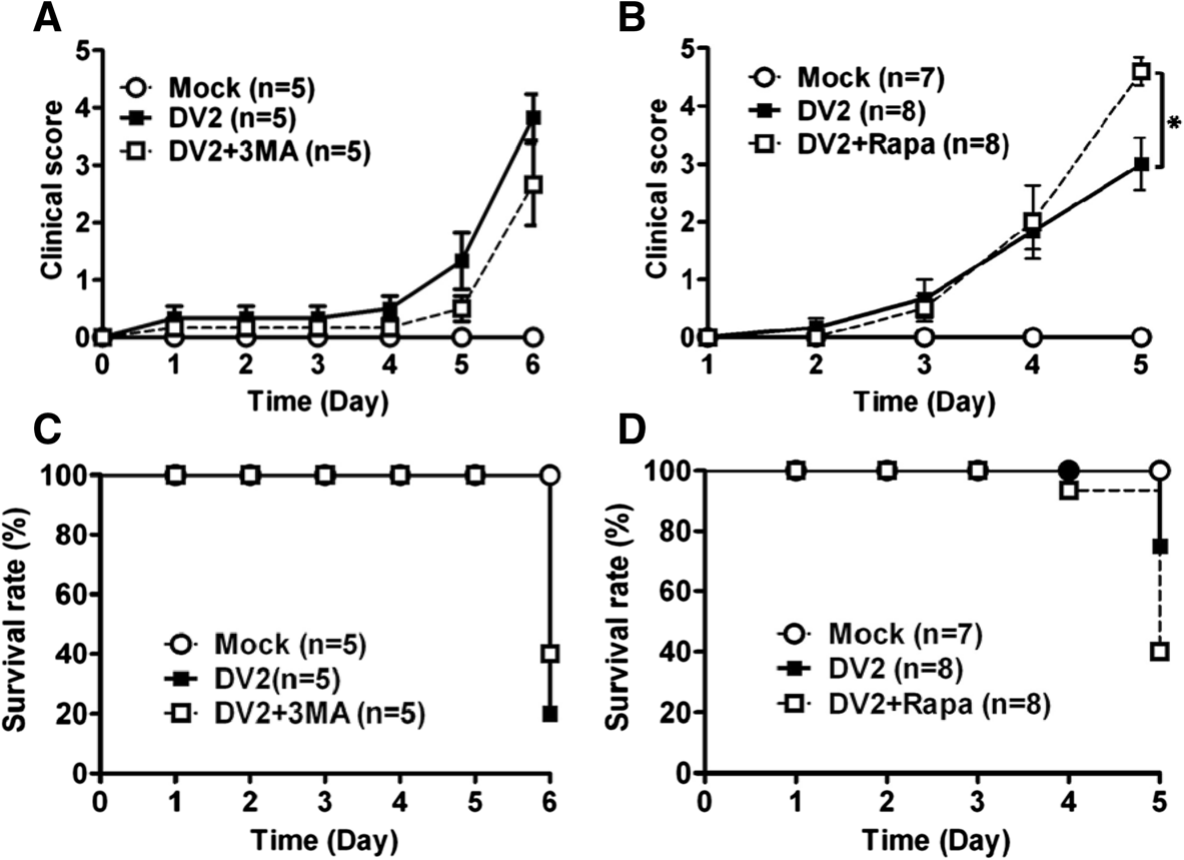
**Manipulation of autophagy by 3-MA or rapamycin affects the clinical score and survival rate of DV2-infected suckling mice.** The six-day-old ICR suckling mice were divided into three groups. The same treatment as in Figure 6 was conducted. **(A)** The clinical score in **(A)** for 3-MA treatment and in **(B)** for rapamacin treatment, as well as survival rate in **(C)** for 3-MA treatment and in **(D)** for rapamycin were monitored daily after the treatments. The criteria of the clinical score are described in Figure 1.

We have demonstrated that 3-MA suppresses autophagy and reduces DV replication *in vitro*[25]. To further investigate the effect of autophagy on DV2-related pathogenesis, mice were pretreated with 3-MA (80μg/g), rapamycin (0.15μg/g), or PBS (Mock) by intracranial injection two hours before DV2 infection. The expression of LC3-II protein was suppressed by about 18% at days 3 and 5 p.i. in the 3-MA treated group (DV2+3MA) as compared to that of the DV2-infected group (Figure 5). The viral titer in the brain of the mice pre-treated with 3-MA was not significantly decreased at days 3 and 5 p.i. (Figure 6A). However, in the group treated with autophagy inducer rapamycin (DV2+Rapa), the viral titer was significantly elevated as compared to that of the DV infection group at day 5 p.i. (Figure 6B). Furthermore, with the addition of 3-MA to block autophagy, the clinical scores were decreased at days 5 and day 6 p.i. in the DV2-infected mice (DV2+3MA) as compared to that of the DV2 infection and mock infection groups (Figure 7A). Accordingly, in the presence of the autophagy inducer rapamycin, the clinical scores of DV2 infection group (DV2+Rapa) were significantly increased at day 5 p.i. compared to those of the DV2 infection and mock infection groups (Figure 7B). The survival rate of DV2-infected mice that received 3-MA treatment was increased to 40% as compared to 20% in the DV2 infection group at day 6 p.i. (Figure 7C). In contrast, the survival rate of the rapamycin-treated group (DV2+Rapa) dropped to 40% as compared to 71% in the DV2 infection group at day 5 p.i. (Figure 7D). Both 3-MA and rapamycin showed no effect on DV2 infection-related loss of body weight in the mice (Additional file 2: A and B). In summary, regulation of autophagy *in vivo* during dengue virus infection could influence physiopathological parameters, including disease symptoms, survival rate, and viral titer.

## Discussion

This study showed that in ICR suckling mice, DV2 can indeed infect the brain tissue at various regions detected by anti-DV-NS1 antibody at five days p.i. (Figure 1D), which is consistent with the results of our previous report that showed DV2 antigen was detected in the brain and liver of the infected mice [38]. However, DV-NS1 antigen was not detected in the cerebellum and pons of the infected mice (Figure 1D). DV2 infection of the brain of 2-day-old Swiss mice induces apoptosis, and dengue virus antigen was detected in the cortical and hippocampal regions [39]. Amaral *et al.* reported that NS3-positive cells could be visualized throughout the parenchyma including the cerebrum, brainstem, and cerebellum in the 6-week-old C57BL/6 mice [40]. The discrepancy between our results and those reported in other studies may be attributed to differences in the strain of mouse or virus, age disparity of the mice, and inoculation titer of the viruses. Taken together, the findings described above indicate that dengue virus could infect various regions of the brain and cause disease symptoms. Our findings are consistent with those of previous reports [39,40].

The results showed that DV2 infection of mouse brain induces autophagy as demonstrated by increased LC3-II protein expression in the brain tissues of DV2-infected mice (Figure 2B), LC3 protein aggregation, and colocalization of DV2-NS1 and LC3 in DV2-infected brain section (Figure 2A), as well as the double-membrane (autophagosome) vesicle formation under TEM (Figure 3C, 3D, and 3E), which was similar to the findings of our previous *in vitro* report [25]. Fusion of autophagosome with endosome to form amphisome was also detected in dengue virus-infected mouse brain (Figure 4), which was consistent with the result of an *in vitro* investigation by Panyasrivanit *et al.* that showed that the endosome harboring dengue viruses fuses with the autophagosome to form amphisome, which serves as the docking site of the viral replication complex [36]. Welsch *et al.* revealed that dengue virus modifies the endoplasmic reticulum (ER) membrane structure to promote its replication and efficient encapsidation of the genome into progeny virus under electron tomography [41]. Miller and Krijnse-Locker reported that viral replication complexes form clusters around the double membrane vesicles, which are formed by contiguous invagination of the ER [42]. The aforementioned reports indicate that DV may replicate at diverse locations, including the autophagosome membrane. Therefore, autophagy may play a role in enhancing viral replication [43]. In this study, we demonstrated the formation of autophagosome and amphisome *in vivo* during DV infection (Figures 2, 3 and 4). Whether these vesicles are also involved in DV replication requires further confirmation.

Autophagy is a dynamic, multi-step process that can be modulated at several steps, both positively and negatively. An accumulation of autophagosomes (measured by TEM, as fluorescent GFP-LC3 dots, or as LC3II lipidation on a Western blot), could reflect either increased autophagosome formation due to increases in autophagic activity, or to reduced turnover of autophagosomes. The latter can occur with the inhibition of their maturation to amphisomes or autolysosomes, which happens if there are defects in the fusion with endosomes or lysosomes, respectively, or following inefficient degradation of the cargo once fusion has occurred [44]. In our study, we demonstrated that the fusion of autophagosome and endosome occurred to form amphisome *in vivo* during DV infection together with p62 degradation, indicating that DV2 infection induces the autophagy flux *in vivo* (Figures 4 and 5). This funding is consistent with our previous *in vitro* report [25].

It has been widely reported that the infection of many viruses affects autophagic flux. Coxsackievirus B3 (CVB3) induces the formation of autophagosomes without promoting lysosome-mediated protein degradation [27,45]. In contrast, HIV-1, Influenza A virus, and HCV infection impair autophagic flux [45]. HCV does not eliminate long-lived protein through autophagic degradation [45,46]. We have demonstrated that DV2 infection triggers autophagic flux and DV2-induced autophagosome is favorable for viral replication in hepatoma cells [25]. Panyasrivanit *et al.* further showed that DV2 titer is increased by blocking autophagic flux using fusion blocker L-asparagine, suggesting that the autophagic flux process decreases DV2 titer [36].

Some viral proteins regulating autophagic activity have been reported. Overexpression of the hepatitis B virus X gene (HBx) enhances starvation-induced autophagy through the upregulation of Beclin 1 expression [47]. Poliovirus 2BC and 3A proteins regulate LC3 modification and membrane induction [30,48]. Furthermore, DV2 NS4A protein induces LC3 cleavage and translocation in epithelial cells [35]. However, whether DV2 NS4A alone *in vivo* is capable of inducing autophagy requires further investigation.

We also demonstrated that expression of Beclin 1 was not changed either in DV-infected or in mock-infected mice (Additional file 1). This result is consistent with the finding of our previous *in vitro* study [25]. Nevertheless, whether Beclin 1 participates in DV-mediated autophagy needs further investigation. We further demonstrated that manipulation of autophagy affects DV2 infection-related pathogenesis, including disease symptoms, survival rate, and virus titer by the autophagy inhibitor, 3-MA, which suppresses the PI3K class III signaling pathway and autophagic activity, and the autophagy inducer rapamycin, which blocks the mTOR signaling pathway. Although encephalitis and neuronal involvement in dengue virus infection are rare, this suckling mice model provides a unique system to clarify dengue-mediated autophagy *in vivo*. While the autophagy inhibitor 3-MA suppressed LC3II expression, it could not significantly suppress virus titer at days 3 and day 5 p.i. *in vivo* (Figures 5 and 6A). It is known that 3-MA plays dual roles in autophagy [49]. Under starvation conditions, 3-MA suppresses PI3K class III and inhibits autophagy; however, under normal conditions, 3-MA promotes autophagic flux. Therefore, the treatment conditions of 3-MA needed to be further optimized to increase its inhibitory effect on autophagy. In summary, manipulation of autophagy by 3-MA and rapamycin affects clinical scores, survival rate, as well as DV2 titer *in vivo*. A recent report demonstrated that DV-NS4A expression induces autophagosome formation during dengue virus infection, and promotes the infected cells to avoid apoptotic cell death, which thus contributes to viral replication [35]. Therefore, whether suppression of DV-mediated autophagy by 3-MA induces infection-mediated apoptosis and further reduces the replication of dengue virus requires further confirmation.

## Conclusions

We demonstrated herein that DV2 infection can induce autophagy both *in vitro*[25] and in *vivo*. Furthermore, manipulation of autophagy by 3-MA or rapamycin affects the replication of DV2 and symptoms of dengue infection *in vivo*. Our results suggest that the DV2-related pathogenesis and survival rate of the suckling mice were enhanced by autophagy, possibly by promoting viral replication.

## Competing interests

The authors declare that they have no competing interests.

## Authors’ contributions

YRL initiated this project and wrote the manuscript. HYH and SHK executed the experiments. HYL, YSL, TMY and CCL conceived the plan. HSL initiated this project and proposed the fundamental frame of this project. All authors read and approved the final manuscript.

## Supplementary Material

Additional file 1 **Beclin 1 expression was not changed in the brain tissue of DV2-infected suckling mice.** Six-day-old ICR suckling mice were intracranially inoculated with DV2 (2.5×10^5^ pfu/mouse) or control media (Mock). After virus infection, mice were sacrificed and the brain tissues were harvested at days 3, 5, and 6 p.i. The expression of Beclin 1 was determined by (A) Western blotting and (B) IHC staining of day 5 brain sections using anti-Beclin 1 antibidy. β-actin was used as the internal control. Click here for file

Additional file 2 **Autophagy inhibitor 3-MA and inducer rapamycin had no effect on the body weight of DV2-infected suckling mice.** Three groups of six-day-old ICR suckling mice were mock-infected or infected by DV2 (2.5 x 10^5^ pfu/mouse) inoculation. At 24 h p.i., mice were treated with 3-MA (80μg/g) in (A), rapamycin (0.15μg/g) in (B), or PBS by intracranial inoculation. The body weight of the mice was determined every day for 5 to 6 days. Click here for file
