# Supplementary figures and images for: Dengue virus infection induces autophagy: an in vivo study
Source: J Biomed Sci. 2013 Sep 8;20(1):65. doi: 10.1186/1423-0127-20-65 (PMC3848819; doi:10.1186/1423-0127-20-65)

**A**

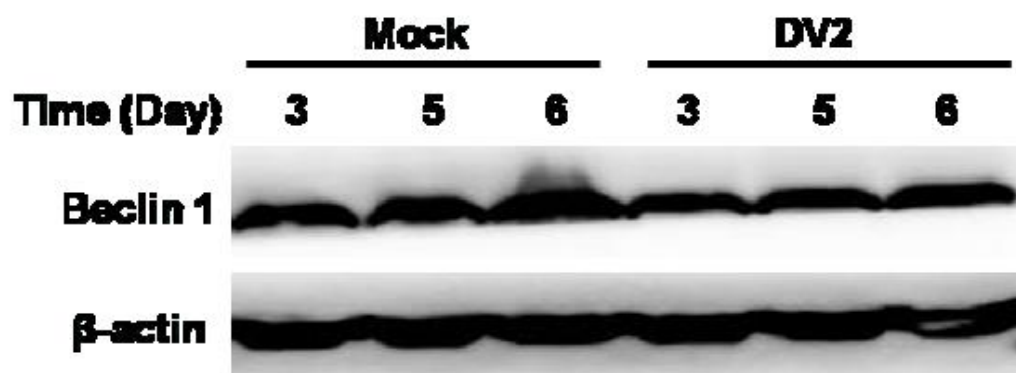

**B**

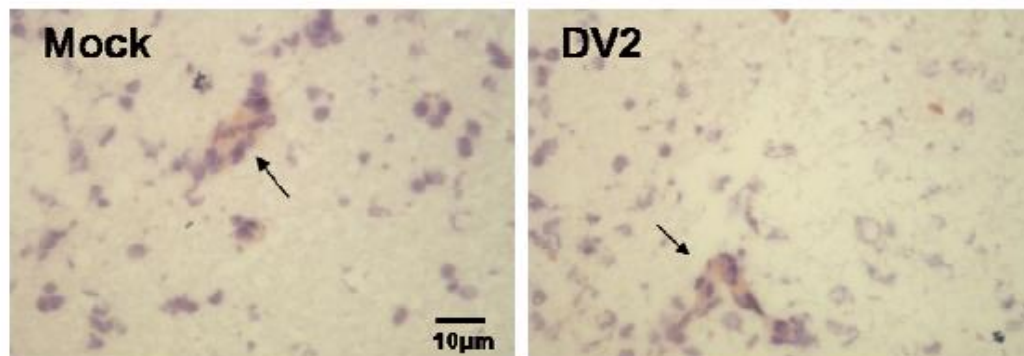

**Additional file 1**

Supplement: Additional file 1 — Beclin 1 expression was not changed in the brain tissue of DV2-infected suckling mice. Six-day-old ICR suckling mice were intracranially inoculated with DV2 (2.5×10^5^ pfu/mouse) or control media (Mock). After virus infection, mice were sacrificed and the brain tissues were harvested at days 3, 5, and 6 p.i. The expression of Beclin 1 was determined by (A) Western blotting and (B) IHC staining of day 5 brain sections using anti-Beclin 1 antibidy. β-actin was used as the internal control. [file 1423-0127-20-65-S1.pdf]

**A**

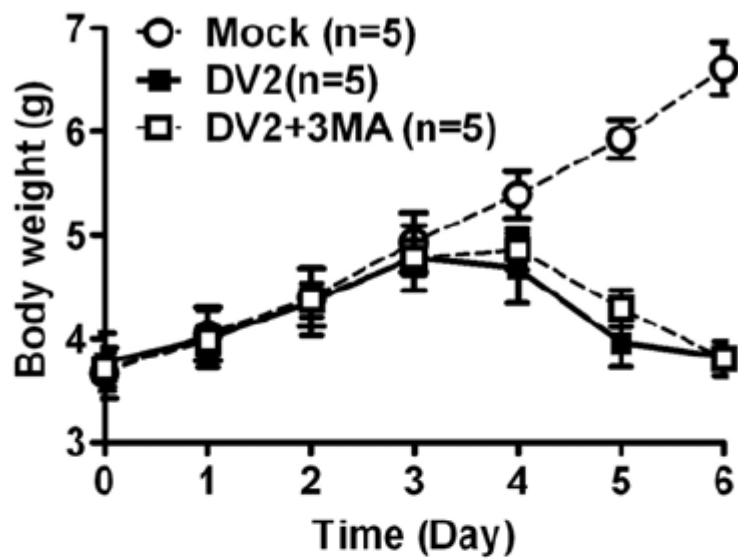

**B**

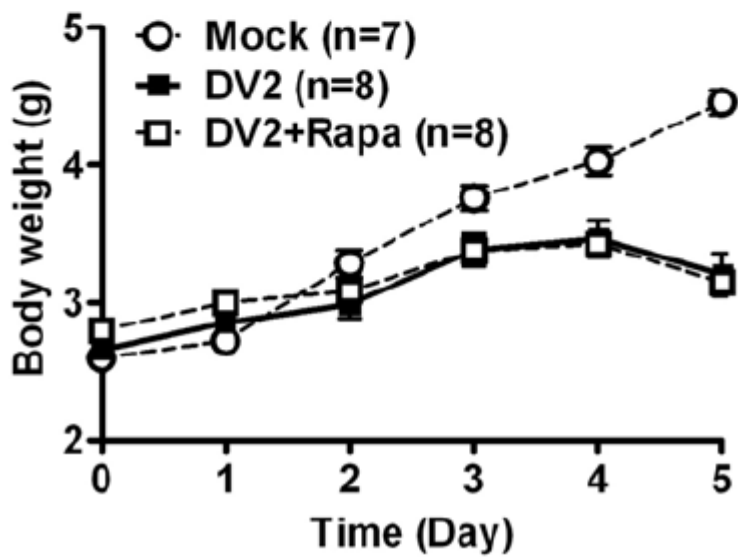

Additional file 2

Supplement: Additional file 2 — Autophagy inhibitor 3-MA and inducer rapamycin had no effect on the body weight of DV2-infected suckling mice. Three groups of six-day-old ICR suckling mice were mock-infected or infected by DV2 (2.5 x 10^5^ pfu/mouse) inoculation. At 24 h p.i., mice were treated with 3-MA (80μg/g) in (A), rapamycin (0.15μg/g) in (B), or PBS by intracranial inoculation. The body weight of the mice was determined every day for 5 to 6 days. [file 1423-0127-20-65-S2.pdf]
